# Supplementary material for: Chromothripsis during telomere crisis is independent of NHEJ, and consistent with a replicative origin
Source: Genome Res. 2019 May;29(5):737–49. doi: 10.1101/gr.240705.118 (PMC6499312; doi:10.1101/gr.240705.118)
Supplement: Supplemental Material [file supp_gr.240705.118_Supplemental_file_1.zip › contigs/annotated_contigs/DB83/contig.2.DB83_length_536_mean_cov_5.03731343284.docx]

**DB83_length_536_mean_cov_5.03731343284**

```
GCACACACCTGAGGTGGGAGGATCGCTGAAGCCCAGGAGTTAGAGGCTACAGTGAATTATGATCGCACCACGGCACTCCAGCCTGCGTG
 >chr17:29884783-29885039 - E=2e-142
ACAGAGGGAGACCCTGTCTCTAAACTTCACTTTGGTAAGGGTTGAGTGGTATATCTGGTTTATCTGATCCCCACATCGGGATCCCTGGG

TAGGACATGTCCAGGAGCTGGTGCTCCCAGGAGTGCGTAGCTGGGGGAGGGATGGCGGCCAAGCGCTGGCAGAGAT|GC|TTCATGGTA
                                                                             >chr17:299
ACCTTGGGAACGAACAGGCTATAATCATGGTGAAAGTGAGCAGTCTTCCAGCTACTGAAGGGGTTACAACAGGGAATGGCACTCTATCA
49340-29949616 - E=2e-154
AAGCCCCATTCCCAGAAAATTGTCCTACTTGAACTGTCTCTCAGTGCCCTGGAAAACCCCACTTGCAAGGCTCTCTTTATTTGACCTGA

TGTAGAACTTGCTCAGTACAAAAAGCCTTTTTCCTGAGGGCATTTGTCAAAAATAATCAGTAGCTATTGTTTATTATTATTATTATT|T

TGAGA
```
